# A Distinct Metabolite Signature in Military Personnel Exposed to Repetitive Low-Level Blasts

**DOI:** 10.3389/fneur.2022.831792

**Published:** 2022-04-07

**Authors:** Michael R. Miller, Alicia DiBattista, Maitray A. Patel, Mark Daley, Catherine Tenn, Ann Nakashima, Shawn G. Rhind, Oshin Vartanian, Maria Y. Shiu, Norleen Caddy, Michelle Garrett, Doug Saunders, Ingrid Smith, Rakesh Jetly, Douglas D. Fraser

**Affiliations:** ^1^Lawson Health Research Institute, London, ON, Canada; ^2^Department of Pediatrics, Western University, London, ON, Canada; ^3^Children's Hospital of Eastern Ontario Research Institute, Ottawa, ON, Canada; ^4^Neurolytix Inc., Toronto, ON, Canada; ^5^Department of Computer Science, Western University, London, ON, Canada; ^6^The Vector Institute for Artificial Intelligence, Toronto, ON, Canada; ^7^Defence Research and Development Canada, Suffield Research Centre, Medicine Hat, AB, Canada; ^8^Defence Research and Development Canada, Toronto Research Centre, Toronto, ON, Canada; ^9^Faculty of Kinesiology & Physical Education, University of Toronto, Toronto, ON, Canada; ^10^Department of Psychology, University of Toronto, Toronto, ON, Canada; ^11^Canadian Forces Health Services, National Defence Headquarters, Ottawa, ON, Canada; ^12^Department of Psychiatry, Faculty of Medicine, University of Ottawa, Ottawa, ON, Canada; ^13^Department of Psychiatry, Faculty of Medicine, Dalhousie University, Halifax, NS, Canada; ^14^Clinical Neurological Sciences, Western University, London, ON, Canada; ^15^Physiology and Pharmacology, Western University, London, ON, Canada

**Keywords:** military, blast, mild traumatic brain injury, metabolites, biomarkers

## Abstract

Military Breachers and Range Staff (MBRS) are subjected to repeated sub-concussive blasts, and they often report symptoms that are consistent with a mild traumatic brain injury (mTBI). Biomarkers of blast injury would potentially aid blast injury diagnosis, surveillance and avoidance. Our objective was to identify plasma metabolite biomarkers in military personnel that were exposed to repeated low-level or sub-concussive blast overpressure. A total of 37 military members were enrolled (18 MBRS and 19 controls), with MBRS having participated in 8–20 breaching courses per year, with a maximum exposure of 6 blasts per day. The two cohorts were similar except that the number of blast exposures were significantly higher in the MBRS, and the MBRS cohort suffered significantly more post-concussive symptoms and poorer health on assessment. Metabolomics profiling demonstrated significant differences between groups with 74% MBRS classification accuracy (CA). Feature reduction identified 6 metabolites that resulted in a MBRS CA of 98%, and included acetic acid (23.7%), formate (22.6%), creatine (14.8%), acetone (14.2%), methanol (12,7%), and glutamic acid (12.0%). All 6 metabolites were examined with individual receiver operating characteristic (ROC) curve analyses and demonstrated areas-under-the-curve (AUCs) of 0.82–0.91 (*P* ≤ 0.001) for MBRS status. Several parsimonious combinations of three metabolites increased accuracy of ROC curve analyses to AUCs of 1.00 (*P* < 0.001), while a combination of volatile organic compounds (VOCs; acetic acid, acetone and methanol) yielded an AUC of 0.98 (*P* < 0.001). Candidate biomarkers for chronic blast exposure were identified, and if validated in a larger cohort, may aid surveillance and care of military personnel. Future point-of-care screening could be developed that measures VOCs from breath, with definitive diagnoses confirmed with plasma metabolomics profiling.

## Introduction

Blast exposure is common in military service (1, 2). A blast wave is generated by an explosion, resulting in a sudden increase in air pressure that is followed by negative pressure, or suction of the blast wave (3). The injury magnitude of a blast wave depends on multiple variables, including the peak of the initial positive-pressure wave, the duration of the overpressure, the medium of the explosion, the distance from the incident blast wave, and the degree of focusing due to a confined area or walls (4). Military breachers and range staff (MBRS) are routinely exposed to repetitive low-level blasts of 2–3 pounds per square inch (psi; or 14–21 kPa) during training and deployment (5–8). Larger blast exposure in the range of 60–80 psi (414–522 kPa) are considered lethal (9).

While the entire body is susceptible to blast injury, the brain seems particularly vulnerable (4). A blast wave not only reflects off the skull, but the generated energy of the shock wave is also absorbed by the brain tissues (3). The kinetic injury from thoracoabdominal compression can also result in transmitted forces to the brain via blood vessels. Regardless of the mechanism, post-blast neurocognitive deficits have been demonstrated in animal models and humans (10, 11). Repetitive low-level blasts, while unlikely to cause mechanical trauma via acceleration and/or rotation of the head, nevertheless result in neuropsychological and neurocognitive deficits and generally decreased physical and mental health (7, 12, 13).

Animal models of blast injury have suggested metabolic impairments that include altered glucose metabolism, associated with a shift from aerobic to anaerobic metabolic pathways. Indeed, increased lactate/pyruvate ratio has been reported (14), followed by decreased energy reserve (14, 15), oxidative stress (16) and inflammation (16–20). The alterations in energy metabolism suggest that blast investigations would benefit from metabolomic profiling, or the measurement of a subject's small metabolite profile, including amino acids, acylcarnitines, glycerophospholipids, sphingolipids, sugars and volatile organic compounds (VOC) (21). Two complementary analytical methods for metabolomics are proton nuclear magnetic resonance (^1^H NMR) spectroscopy and mass spectrometry (MS), yielding measurement accuracies in the μM and pM ranges, respectively.

As the consequences of long-term exposure to repetitive low-level blasts is largely unknown, as are the injury thresholds, or when blast exposure initiates poorer health and compromised well-being, identification of accurate blast biomarkers and assays is critical to understanding blast exposure pathophysiology. Thus, the aims of this study were (1) to profile two Canadian Armed Forces (CAF) military cohorts (MBRS and non-MBRS) with metabolomics, (2) to identify novel metabolite biomarkers of MBRS with machine learning; and (3) to correlate biomarkers with blast injury symptoms.

## Methods

The study protocol was approved by the Human Research Ethics Committee of Defense Research and Development Canada. Potential participants were recruited via an electronic recruitment poster that was circulated among the Canadian Forces School of Military Engineering (CFSME) staff (MBRS) and at Denison Armory (for non-MBRS or controls) (7). Blast quantification was not attempted; however, the instructors and range staff potentially contribute to eight-twenty breaching courses per year with one-two days of breaching on the range. The instructors and range staff form a “cell” that administer the courses together for a period of one-three years; while they may be exposed to more than six blasts per day, the magnitude and number of blast events varies.

All data were collected in a single session for each participant. CFSME MBRS were tested at Canadian Forces Base Gagetown. Sex- and age-matched CAF controls were tested at DRDC TRC (Toronto Research Center). The measures included neuropsychological and neurocognitive tasks, as well as blood procurement for biomarker studies. Specifically, participants completed a demographic and service history survey, a Background Health Questionnaire, the RAND SF-36 Health Survey, the Short Musculoskeletal Function Questionnaire (SMFA), a modified version of the Rivermead Post-Concussion Symptoms Questionnaire and a Post-Traumatic Checklist (PCL-5) (please see Supplementary Table 1 for additional information on each survey) (22–26).

All blood samples were collected via strict standard operating procedures. The participants had not been exposed to blast for a minimum of 48 h and were asked to refrain from strenuous physical activity for at least 24 h prior to testing. Peripheral blood was collected by venipuncture from participants at rest and in a fasting state by a trained technologist using standard phlebotomy techniques. Venous blood samples were drawn into 10-mL EDTA tubes, immediately centrifuged at 1,600 × g for 15 min at 4°C, separated into plasma aliquots, and stored at −80°C until analysis. All samples were processed in the same manner at the same time of day by the same technologist(s). A targeted quantitative approach was applied to analyze plasma samples using both a ^1^H NMR and a combination of direct injection tandem MS (DI-MS/MS) and liquid chromatography tandem MS (LC-MS/MS) using the AbsoluteIDQ p180 kit (Biocrates Life Sciences, AG, Innsbruck, Austria), as previously described (27, 28). In the event of metabolite repeats measured with both techniques, the ^1^H NMR metabolite measurements were deleted from the combined metabolite database and only DI-MS data analyzed.

For feature selection and classification accuracy, the raw data for each subject were ingested within each feature, across subjects. A random forest classifier was trained on the variables to predict MBRS status (“scikit-learn” module for Python 3.8.5 Open Source). A random forest is a set of decision trees, and consequently, we were able to interrogate this collection of trees to identify the features that had the highest predictive value. Feature selection was not performed in preprocessing. During training, the random forest classifier performed an implicit feature selection; the top features were those that appear highest ranked in the most trees. To reduce overfitting, the number of trees and maximum depth of each tree was limited (29); thus, MBRS status was determined using a 3-fold cross validation with a random forest of ten trees and maximum depth of three. To remain conservative and to further limit the risk of overfitting, no hyperparameters were tuned or optimized by design and intent. For feature selection, the samples were split into status-stratified training (70%) and validation sets (30%). A Boruta feature selection method, based on random forest classifiers, was then used to develop a reduced model using the training dataset (30). The reduced metabolite dataset was then visualized with a non-linear dimensionality reduction on the full data matrix using the t-distributed stochastic nearest neighbor embedding (t-SNE) algorithm (31). t-SNE assumes that the “optimal” representation of the data lies on a manifold with complex geometry, but with low dimension, embedded in the full-dimensional space of the raw data.

Medians (IQRs) and frequency (%) were used to report continuous and categorical variables, respectively. Continuous variables were compared using Mann-Whitney U tests, and categorical variables were compared using chi-square tests (or Fisher's exact chi-square, as appropriate). Receiver operating characteristic (ROC) curves were estimated for individual metabolites and continuous outcomes in terms of predicting MBRS status, with area-under-the-curve (AUC) >0.7 considered acceptable. The coordinates of the curves for individual metabolites were analyzed using Youden's index to identify cut-off values in μM based on the highest sensitivity and specificity for predicting MBRS status. Metabolite combinations were calculated through logistic regression models with MBRS status as the outcome and the representative metabolites as the included predictors; the predicted values from the regression models were then saved for use in ROC curve analyses. All analyses were conducted using SPSS version 27 (IBM Corp., Armonk, NY, USA), and *p*-values <0.05 were considered significant. Heat maps depicting Pearson correlation values between metabolites and outcomes were created in R (http://www.r-project.org) using the ggplot2 version 3.3.3 package (32).

## Results

We prospectively included nineteen MBRS and nineteen age- and sex-matched non-MBRS (Table 1); the final MBRS cohort number was eighteen as there was insufficient plasma from one service member for analyses. Nonetheless, the two cohorts were well-balanced for age, sex, education, military status, lifestyle and injuries. The MBRS cohort was more likely to be francophone, senior in rank, had a longer duration of service and combat deployed. As expected, MBRS were exposed to a significantly greater number of blasts.

**Table 1:** Military personnel demographics, service history and injuries/exposures.

**Variable**	**Non-MBRS (*n* = 19)**	**MBRS (*n* = 18)**	***P*-value**
Age (yrs), med (IQR)	32 (27, 36)	32 (26, 38)	0.976
Male sex, n (%)	17 (90)	16 (89)	>0.994
Height (cm), med (IQR)	179 (173, 188)	179 (177, 183)	0.867
Weight (lbs), med (IQR)	188 (170, 200)	180 (168, 215)	0.855
Body mass index	25.1 (24.4, 28.8)	26.6 (23.8, 30.1)	0.704
Education, *n* (%)			0.633
High school	4 (21)	6 (33)	
College	4 (21)	6 (33)	
Undergraduate university	10 (53)	5 (28)	
Graduate university	1 (5)	1 (6)	
**First language**, ***n*** **(%)**			0.001^*^
English	16 (84)	11 (61)	
French	0 (0)	7 (39)	
Other	3 (16)	0 (0)	
Military status, *n* (%)			0.630
Forces	8 (42)	9 (50)	
Reserves	11 (58)	10 (53)	
**Rank**, ***n*** **(%)**			<0.001^*^
Junior NCM	13 (68)	5 (28)	
Senior NCM	0 (0)	11 (61)	
Junior officer	6 (32)	2 (11)	
**Years of service, med (IQR)**	5 (1, 11)	11 (9, 14)	0.005^*^
Allergies, *n* (%)	6 (32)	3 (17)	0.447
Medications, *n* (%)	5 (26)	5 (29)	>0.994
Coffee/caffeine drinks/day, med (IQR)	1 (1, 2)	2 (1.4, 3)	0.054
Alcoholic drinks/week, med (IQR)	2 (1, 5)	2.8 (1, 8.5)	0.384
Smoke, *n* (%)	2 (11)	3 (17)	0.660
Use drugs in last 6 mo, *n* (%)	0 (0)	1 (6)	0.472
Current cold/infection, *n* (%)	0 (0)	3 (17)	0.105
Exercise regularly, *n* (%)	18 (95)	15 (88)	0.593
Specific diet, *n* (%)	3 (16)	2 (11)	>0.994
**Injuries and exposures**, ***n*** **(%)**			
**Combat deployment**	0 (0)	10 (63)^a^	<0.001^*^
Concussion	5 (26)	8 (47)	0.196
Head impact	11 (58)	9 (50)	0.630
Motor vehicle collision	9 (47)	14 (78)	0.057
Fall as a child	6 (32)	8 (44)	0.420
Physical fight	15 (79)	12 (67)	0.476
**Blast exposure**	2 (11)	18 (100)	<0.001^*^
**Years breaching**	0 (0.0)	6.5 (3.8, 10.0)	<0.001^*^
**Years explosives**	0 (0.0)	10.0 (6.8, 12.0)	<0.001^*^

*MBRS, military breachers/range staff. Continuous variables are presented as median (IQR), and categorical variables are presented as n (%)*.

a *Data unavailable for 2 MBRS members*.

* *p < 0.05*.

*Bold indicate statistical significance*.

The reported symptoms and health assessment data are listed in Table 2. The MBRS cohort reported an increased number of symptoms, with reduced general physical and mental health. Energy was lower for the MBRS cohort, and functional and emotional health suffered. The Rivermead Post-Concussion Symptoms were worse for MBRSs, including both early and late symptoms, as well as somatic, cognitive and emotional health. Finally, perceived stress was significantly worse for MBRSs.

**Table 2:** Military personnel reported symptoms and health assessment.

**Variable**	**Non-MBRS (*n* = 19)**	**MBRS (*n* = 18)**	***P*-value**
**Reported symptoms**			
Chronic disease, *n* (%)	2 (11)	1 (6)	>0.994
**Headache**	0 (0, 2)	2 (1, 2.3)	0.010^*^
**Dizziness**	0 (0, 1)	1.5 (0, 2)	0.043^*^
Vomiting	0 (0, 0)	0 (0, 0.3)	0.356
Noise sensitivity	0 (0, 0)	0 (0, 2)	0.099
**Sleep disturbance**	0 (0, 0)	0.5 (0, 2)	0.011^*^
**Fatigue**	0 (0, 0)	1.5 (0, 2)	0.002^*^
**Irritable**	0 (0, 0)	1 (0, 2.3)	0.013^*^
**Depressed**	0 (0, 0)	0 (0, 1)	0.003^*^
**Frustrated**	0 (0, 0)	0.5 (0, 3)	0.006^*^
**Forgetful**	0 (0, 0)	1 (0, 2)	<0.001^*^
**Poor concentration**	0 (0, 0)	1 (0, 2)	<0.001^*^
**Taking long to think**	0 (0, 0)	1 (0, 1.3)	0.003^*^
Blurred vision	0 (0, 0)	0 (0, 0.3)	0.137
Double vision	0 (0, 0)	0 (0, 0.3)	0.137
**Light sensitivity**	0 (0, 0)	0 (0, 1)	0.030^*^
**Restlessness**	0 (0, 0)	0 (0, 1)	0.007^*^
**Impaired comprehension**	0 (0, 0)	0 (0, 1)	0.015^*^
Impaired reasoning	0 (0, 0)	0 (0, 1)	0.061
Impaired logic	0 (0, 0)	0 (0, 0.3)	0.131
**General physical health**	5 (4, 5)	4 (3, 4)	0.002^*^
**General mental health**	5 (4, 5)	4 (3.8, 4)	0.037^*^
**RAND SF-36 Health Survey**			
Physical functioning	100 (95, 100)	98 (94, 100)	0.278
Physical limitations	100 (100, 100)	100 (94, 100)	0.866
Emotional limitations	100 (67, 100)	100 (92, 100)	0.346
**Energy**	65 (60, 80)	48 (35, 66)	0.017^*^
Emotional well-being	84 (68, 88)	78 (60, 88)	0.540
Social functioning	100 (75, 100)	100 (81, 100)	0.727
General health	80 (65, 95)	75 (63, 80)	0.285
Pain	90 (80, 100)	90 (79, 93)	0.187
**Short musculoskeletal function assessment (SMFA)**			
**Function**	34.0 (34.0, 41.0)	40.0 (37.0, 47.0)	0.018^*^
Bother	12.0 (12.0, 17.0)	15.0 (12.8, 17.8)	0.092
Daily activities	10.0 (10.0, 10.0)	10.0 (10.0, 11.3)	0.321
**Emotional status**	7.0 (7.0, 13.0)	11.5 (9.0, 16.3)	0.011^*^
Arm and hand	8 (8, 8)	8 (8, 8)	0.323
Mobility	9.0 (9.0, 10.0)	9.0 (9.0, 12.3)	0.577
**Rivermead post concussion symptom questionnaire**			
**RPQ3**	0 (0, 2.0)	2.5 (1.0, 6.0)	0.004^*^
**RPQ13**	0 (0, 3.0)	9.0 (0.8, 17.8)	0.001^*^
**Somatic**	0 (0, 0.2)	0.6 (0, 1.4)	0.006^*^
**Cognitive**	0 (0, 0)	0 (0, 1.5)	0.005^*^
**Emotional**	0 (0, 0)	0 (0, 1.3)	0.003^*^
Post-traumatic stress disorder (PTSD)	0 (0, 9)	6 (0, 11)	0.106

*MBRS, military breachers/range staff. Continuous variables are presented as median (IQR), and categorical variables are presented as n (%)*.

* *p < 0.05*.

*Bold indicate statistical significance*.

**Figure 1:**
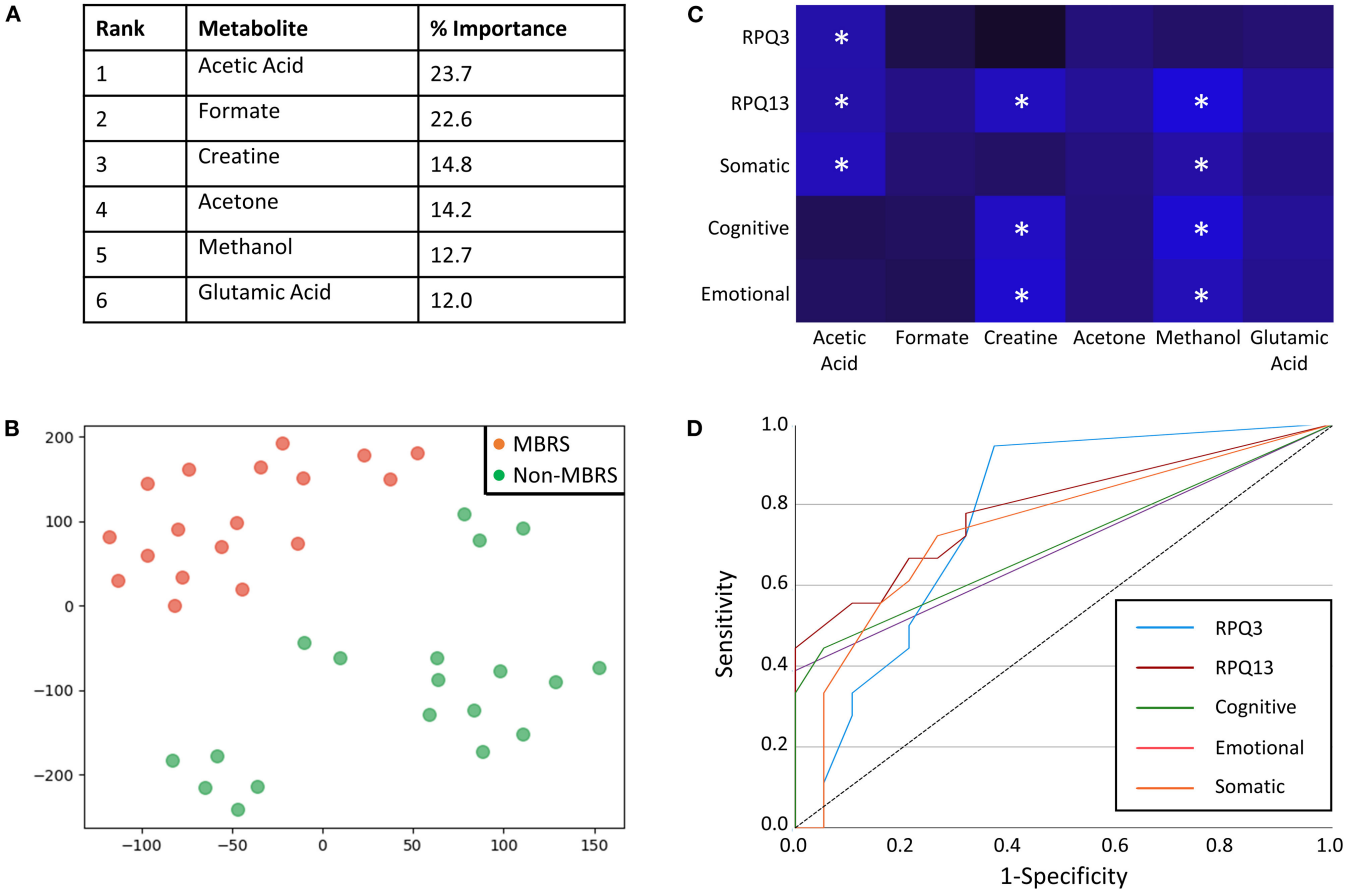
Metabolites identified with feature selection that determine military breacher/range staff (MBRS) status and their relationship with Rivermead post-concussion variables. **(A)** A rank order of six leading plasma metabolites that classify MBRS vs. non-MBRS with 98% classification accuracy. All six metabolites are significantly decreased in plasma from MBRS when compared to non-MBRS. Their relative % importance is shown. **(B)** A tSNE plot demonstrating that MBRS and non-MBRS can be easily separated and identified based on plasma levels of the leading six metabolites. The axes are dimension-less. **(C)** A heat map demonstrating the negative correlations between Rivermead post-concussion variables and plasma levels of the six leading metabolites. Brighter blue represents a stronger negative correlation. Statistically significant negative correlations are indicated with white asterisks (^*^P < 0.05). **(D)** ROC curves illustrating that the Rivermead post-concussion variables are predictive of MBRS status, as well as with the metabolite parsimonious combinations listed in Table 4 [RPQ13 (late symptoms) AUC = 0.79 [0.65–0.94], RPQ3 (early symptoms) AUC = 0.77 [95%CI 0.62–0.93], Somatic AUC = 0.75 [95%CI 0.58–0.91], Cognitive AUC = 0.71 [95%CI 0.53–0.88], and Emotional AUC = 0.69 [95%CI 0.52–0.87].

Metabolomic profiling of cohorts was accomplished with both ^1^H NMR and DI-MS, with a total of one hundred and seventy plasma metabolites measured. Cohort classification accuracy was 74% when the entire metabolome was ingested. Feature selection narrowed the leading metabolites down to six, providing a classification accuracy of 98% (Figure 1A). The six metabolites included acetic acid, formate, creatine, acetone, methanol and glutamic acid, and all six were significantly lower in the MBRS cohort (*P* ≤ 0.001; Table 3). A tSNE plot demonstrated near perfect separation of cohorts based on the leading six metabolites (Figure 1B). The decreased levels of acetic acid, creatine and methanol correlated with increased symptom scores reported on the Rivermead Post-Concussion Symptoms Questionnaire (Figure 1C). ROC curve analyses of the 6 individual metabolites for determining MBRS status demonstrated AUCs of 0.82–0.91 (*P* ≤ 0.001; Table 4). The cut-off values for each metabolite were determined. Scatter plots with metabolite cut-off values are shown for Rivermead early (RPQ3) and late (RPQ13) symptoms, as well as RAND Energy (Supplementary Figures 1–3). We then identified three parsimonious combinations of three metabolites that perfectly predicted MBRS status with AUCs = 1.00 (*P* < 0.001; Table 4). One metabolite combination that consisted of only VOCs, including acetic acid, acetone and methanol, yielded an AUC of 0.98 (*P* < 0.001). The RAND energy level and the Rivermead Post-Concussion Symptom Questionnaire scores predicted both MBRS status and the parsimonious metabolite combinations, yielding AUCs of 0.69–0.79 (Figure 1D; the RAND Energy level not shown, AUC=0.73 [95% CI 0.56–0.90]).

**Table 3:** Military personnel metabolite parameters.

**Metabolite**	**Non-MBRS (*n* = 19)**	**MBRS (*n* = 18)**	**Direction**	***P*-value**
Acetic acid	36.6 (29.5, 43.5)	20.3 (14.5, 26.4)	↓	**<0.001**
Formate	58.1 (55.6, 303.8)	40.3 (38.4, 45.5)	↓	**<0.001**
Creatine	30.1 (25.7, 38.6)	20.7 (17.4, 26.3)	↓	**<0.001**
Acetone	15.8 (11.0, 17.6)	8.7 (7.4, 9.7)	↓	**<0.001**
Methanol	42.3 (28.6, 47.3)	24.8 (22.0, 31.5)	↓	**<0.001**
Glutamic acid	47.5 (37.1, 60.0)	28.2 (23.2, 37.1)	↓	**0.001**

MBRS, military breachers/range staff. Continuous variables are presented as median (IQR). All biochemical values are in μM. ^*^ p < 0.05.

*Bold indicate statistical significance*.

**Table 4:** ROC curve summary predicting MBRS status.

**Predictor(s)**	**AUC (SE)**	**95% CI**	***P*-value**	**Cut-off value**
***Individual metabolites***				
Acetic acid	0.91 (0.05)	0.80–1.00	**<0.001**	<28.26
Formate	0.89 (0.06)	0.77–1.00	**<0.001**	<53.16
Creatine	0.87 (0.06)	0.75–0.98	**<0.001**	<22.80
Acetone	0.90 (0.05)	0.80–1.00	**<0.001**	<10.77
Methanol	0.86 (0.06)	0.74–0.98	**<0.001**	<35.47
Glutamic acid	0.82 (0.08)	0.66 – 0.97	**0.001**	<36.90
***Parsimonious combinations***				
Acetic acid, methanol, glutamic acid	1.00 (0.00)	1.00–1.00	**<0.001**	-
Acetone, methanol, glutamic acid	1.00 (0.00)	1.00–1.00	**<0.001**	-
Creatine, methanol, glutamic acid	1.00 (0.00)	1.00–1.00	**<0.001**	-
***Volatile organic compound combination***				
Acetic acid, acetone, methanol	0.98 (0.00)	0.95–1.00	**<0.001**	-

*MBRS, military breachers/range staff. Receiver operating characteristic (ROC) curves were estimated for individual metabolites and continuous outcomes in terms of predicting Breacher status, with area-under-the-curve (AUC) >0.7 considered acceptable. Combinations were created using predicted values from a logistic regression with Breacher status as the outcome and the metabolite combinations as the predictors. Cut-off values were calculated using Youden's index, and are presented as μM. Bold indicate statistical significance*.

## Discussion

In this study, we performed metabolomics profiling of CAF members, both MBRS and non-MBRS. Our data confirm that MBRS suffer post-concussive symptoms associated with mTBI and poorer health. Reduced metabolite parsimonious combinations identified MBRS status with 100% accuracy, and a combination of three VOCs with 98% accuracy. Our data suggest that repetitive exposure to low-level blasts in military personnel may be potentially identified by measuring as few as three metabolites.

Low-level blasts refer to controlled blast exposures that occur during standard training procedures and some military operations experienced by military personnel (33). While MBRS are frequently exposed to low-level blast overpressure, exposures are also prevalent for any service member firing artillery, mortars, grenades and/or shoulder-fired weapons. Given the exposure frequency, it is not surprising that up to one quarter of military service members experience post-concussive symptoms (1, 2). Exposure to a 4 psi (28 kPa) blast is considered to be minimally harmful; however, these thresholds were established based on tympanic membrane rupture (5, 34) and neglect the cumulative consequences of repetitive exposures to low-level blast events. The military doctrine limits blast exposure to 3 psi (21 kPa); however, these values were often exceeded when military personnel exposed to blasts wore pressure gauges (8, 34).

Metabolomics profiling of CAF members included use of both ^1^H NMR and DI-LC-MS/MS to yield quantitative measurements of 170 metabolites. Feature selection identified the leading 6 metabolites for determining MBRS status, with a 98% classification accuracy. Of the 6 leading metabolites, five are related to energy metabolism (acetic acid, formate, creatine, acetone and methanol), and one is an excitatory amino acid (glutamic acid). Classification accuracy for determining MBRS status increased to 100% with three parsimonious combinations of metabolites, while combining only VOCs resulted in a 98% classification accuracy. Three individual metabolites negatively correlated with Rivermead Post-Concussion Symptoms Questionnaire (acetic acid, creatine and methanol), while RAND Energy and Rivermead early and late symptoms predicted both MBRS status and the three metabolite parsimonious combinations. Our metabolite biomarkers appear to offer a promising complement or alternative to conventional protein biomarker assays for identifying/characterizing blast exposures (6, 35–37).

Acetic acid is absorbed from the gastrointestinal tract and through the lung, or formed as a final product of enhanced β-oxidation of fatty acids. Acetic acid is utilized as fuel in extrahepatic tissues and may give rise to the production of ketone bodies as intermediates. Consumption of acetic acid improves glucose tolerance and insulin sensitivity (38, 39). Supplementation with dietary acetic acid is well-tolerated, has no adverse side effects, and may improve overall energy metabolism (40). In the brain, acetic acid increases tricarboxylic acid cycle flux and neuronal excitability via glutamate neurotransmission (41). As MBRS members had significant reductions in plasma acetic acid, and its levels negatively correlated with mTBI symptoms, it is possible that oral supplementation may aid the low energy and neurocognitive symptoms identified by MBRS.

Creatine facilitates ATP homeostasis during energy turnover and it acts as an antioxidant by attenuating reactive oxygen species (42). In the brain, creatine is also important for energy production via a brain-specific isoform of creatine kinase. Creatine deficiencies result in mental and cognitive derangements (43), which can be partially attenuated by creatine supplementation (44). Creatine supplementation is suggested to aid TBI outcome, and clinical trials on military personnel have been encouraged (45). Indeed, creatine levels in MBRS members negatively correlated with mTBI symptoms.

Formate is an intermediate in one-carbon (1C) metabolism and is produced in a variety of metabolic reactions within cellular compartments, including folate-dependent (e.g., via serine, glycine, methionine, sarcosine and choline catabolism) and folate-independent (e.g., catabolism of tryptophan, methionine salvage, α-oxidation of branched chain fatty acids) reactions (46). Formate can also be produced by anaerobic fermentation by the gut microbiome (47). Fermentation of fruits and vegetables by the microbiome can also produce methanol, which is metabolized to formaldehyde and then formate by the liver. Animal studies have demonstrated that spinal cord injuries cause a reduction in intestinal motility and permeability, leading to alterations in intestinal bacterial composition know as gut dysbiosis, whereas TBI causes intestinal bacterial speciation changes as rapidly as 2 h post-trauma (48). Gut dysbiosis may alter bacterial fermentation-produced circulating methanol and formate levels and thus impact 1C metabolism (including generation of S-adenosylmethionine) (49). Dietary supplementation of 1C sources, including formate, creatine, choline and betaine, may help restore 1C metabolism and possibly ameliorate symptoms of mTBI.

Acetone, together with acetoacetate and beta-hydroxybutyrate, are the ketone by-products of fat metabolism in the liver (50). Acetoacetate is formed from acetyl-CoA, and then beta-oxidized to 3-beta-hydroxbutyrate. When required for energy production, acetoacetate is converted back to acetyl-CoA to be incorporated into the TCA cycle. Decarboxylation of excess acetoacetate produces acetone, which cannot be used for energy production directly and is either exhaled or excreted as waste. However, acetone can also be converted into lactic acid within the liver, which is then subsequently oxidized into pyruvic acid. The latter can also produce acetyl-CoA to be incorporated into the TCA cycle. The decreased plasma acetone levels measured in MBRS members may reflect less acetoacetate decarboxylation and/or greater acetone conversion to pyruvic acid, to compensate energy deficits. In addition, elevating plasma ketones via dietary manipulation (i.e., carbohydrate restriction) may improve blast-induced symptoms (51). Indeed, ketones are actively transported into brain via monocarboxylate transporters, and up to two-thirds of brain metabolism can be fueled by ketones.

Glutamic acid is a non-essential amino acid that is a major mediator of excitatory signals in the brain and is involved in most aspects of normal brain function including cognition, memory and learning (52). Measurements of glutamic acid in plasma are generally thought to reflect brain levels, as there are no glutamate degrading enzymes and regulation of glutamic acid levels is controlled via cellular release and cellular uptake (53, 54). High plasma glutamic acid levels are associated with acute/sub-acute TBI, as well as anxiety, autism, bipolar disorder, depression, impulsivity and stroke. Low plasma glutamic acid levels are often attributed to ammonia toxicity, and more recently, to Parkinson's disease (55). The low levels of glutamic acid found in plasma from MBRS members may represent a relative exhaustion of glutamate production after chronic low-level blast exposure, and separates itself from the excessive glutamate release and subsequent excitotoxicity suffered acutely after mechanical TBI. It is also possible that a delayed disruption of excitatory glutamate circuits may underlie the deficits in cognitive and motor function reported by MRBS members. Finally, as glutamate clearance from brain to blood occurs via gradient transport across the blood brain barrier, blood glutamate scavenger mechanisms could be upregulated, and may indirectly reflect ongoing brain injury or healing mechanisms.

There is significant novelty and potential use of these data. We report that a small number of metabolites can potentially determine whom has been chronically exposed to low-level blasts, and that the measured decreases in plasma metabolites correlate with increased mTBI symptoms. Our data are supported by the findings of our co-authors who demonstrated that putative neurological biomarkers (S100beta, GFAP, UCH-L1, pNF-H, and T-tau) were elevated in this chronic blast exposed military cohort (Published Abstract; Rhind et al., Journal of Neurotrauma, 2018, A99). Furthermore, a combination of VOCs can be accurately measured with portable, hand-held breathalyzers, and may be used as future point-of-care monitoring devices for service members in both training and theater. A laboratory quantitative test can be easily developed that would require analysis of a blood sample with either ^1^H NMR or quantitative mass spectrometry. Indeed, the latter approach is currently under clinical testing for concussion diagnostics, whereby the pattern of metabolite change is unique and primarily lipidomic (56). As the metabolite profile for repetitive blast exposure is unique, it is possible that metabolite signatures will be useful for separating various forms of trauma, including blast, mechanical, neurochemical and psychological.

Our study was not without limitations. First, the number of subjects investigated were limited and validation is required in a larger cohort combined with brain-specific measurements. Second, we cannot rule out that some metabolic changes may be driven by chronic blast injury to peripheral organs. Third, the metabolite patterns may not be generalizable to non-MBRS or non-CFSME military members. Fourth, it is unclear if all blasts, including repetitive high-caliber rifle fire and improvised explosive device, would result in similar metabolite changes. Fifth, the contribution of combat deployment to metabolite changes in the MBRS cohort is unclear. Sixth, while the accurate cut-off values for each plasma metabolite were established, they require validation as injury warning thresholds. Despite these caveats, our data suggest that MBRS can be monitored for cumulative blast injury with both quantitative laboratory approaches (^1^H NMR and/or GC-MS/MS), and with future point-of-care screening (e.g., breath VOCs).

## Conclusions

We report a distinct metabolite signature in military personnel suffering post-concussive symptoms and associated poorer health, following exposure to repetitive low-level blasts. Reduced plasma metabolite combinations, which were associated with energy metabolism and an excitatory amino acid neurotransmitter, identified MBRS status with 100% accuracy. A combination of three VOCs identified MBRS status with 98% accuracy. Repetitive blast exposure may be accurately identified in military personnel with as few as three metabolites. Our data also suggest that a future point-of-care screening test could be developed that measures VOCs in breath. The metabolite biomarkers for blast exposure identified here may aid blast injury surveillance and the care and well-being of military personnel. Oral supplementation with acetic acid, creatine and 1C sources, as well as carbohydrate restriction (i.e., ketosis), may alleviate some blast-induced symptoms related to brain energy metabolism, but support for these interventions would require a rigorous randomized controlled trial.

## Data Availability Statement

The original contributions presented in the study are included in the article/Supplementary Material, further inquiries can be directed to the corresponding author/s.

## Ethics Statement

The studies involving human participants were reviewed and approved by Human Research Ethics Committee of Defence Research and Development Canada. The patients/participants provided their written informed consent to participate in this study.

## Author Contributions

DF: concept, methods design, data collection, data analysis, data interpretation, manuscript writing, and submission. MM, MP, MD, CT, SR, and OV: data collection, data analysis, and manuscript writing. AD, AN, MS, NC, MG, DS, IS, and RJ: data collection and critical review of the manuscript. All authors contributed to the article and approved the submitted version.

## Funding

This research was supported by funding from the Department of National Defence (DND) and the Canadian Forces Health Services.

## Conflict of Interest

DF discloses a provisional patent and the licensing of technology to Neurolytixs Inc., a diagnostics biotechnology company that is focused on mild traumatic brain injury (www.neurolytixs.com). AD and DF are members of Neurolytixs Inc. The remaining authors declare that the research was conducted in the absence of any commercial or financial relationships that could be construed as a potential conflict of interest.

## Publisher's Note

All claims expressed in this article are solely those of the authors and do not necessarily represent those of their affiliated organizations, or those of the publisher, the editors and the reviewers. Any product that may be evaluated in this article, or claim that may be made by its manufacturer, is not guaranteed or endorsed by the publisher.
