# Supplementary material for: A Distinct Metabolite Signature in Military Personnel Exposed to Repetitive Low-Level Blasts
Source: Front Neurol. 2022 Apr 7;13:831792. doi: 10.3389/fneur.2022.831792 (PMC9021419; doi:10.3389/fneur.2022.831792)
Supplement: Supplementary file 1 [file Table_1.DOCX]

**Supplemental Table 1. Survey measures used in study**

The *RAND SF-36 Health Survey* has 36 items aggregated into 8 health-related categories, where a score of 100 indicates optimal functioning in each category (Physical functioning, Physical limitations, Emotional limitations, Energy, Emotional well-being, Social functioning, General health, and Pain) (Ware & Sherbourne, 1992).

The *Rivermead Post Concussion Symptoms Questionnaire (RPQ)* is a measure of symptoms commonly experienced after head injury (King et al., 1995). The RPQ can be scored and analyzed using two methods: The first method involves separating its items into two clusters that capture either the 3 “early” post-concussive symptoms that present themselves closer to the time of injury, including headache and feelings of dizziness (i.e., RPQ-3), or 13 “late” post-concussive symptoms that present themselves later following the injury, such as sleep disturbance and sensitivity to noise (i.e., RPQ-13) (Eyres et al., 2005). The second method involved separating RPQ’s items into clusters that capture cognitive (e.g., poor concentration), emotional (e.g., being irritable), or somatic (e.g., fatigue) symptoms associated with concussion (Verfaellie et al., 2013). Our participants completed a modified version of the RPQ, where for each symptom they were asked to indicate whether they had experienced it as a function of injury to the head. We calculated RPQ scores using both scoring methods described above, and have also provided item-specific analyses for a comprehensive analysis of the results.

Symptomatic criteria for PTSD were assessed using the 20-item PCL-5, according to the Diagnostic and Statistical Manual of Mental Disorders (DSM-5) (Weathers et al., 2015).

The *Short Musculoskeletal Function* Questionnaire (SMFQ; Swiontkowski et al., 1999) generates scores on two indices: The *Dysfunction Index* (DI) assesses the participant’s perceptions of his or her functional musculoskeletal performance, whereas the *Bother Index* (BI) assesses how much the participant is bothered by musculoskeletal problems.

In addition to the measures above, we also asked our participants to rate themselves on the following five individual items: Impaired comprehension, Impaired reasoning, Impaired logic, General physical health, and General mental health. The first three items were included to obtain additional insights into the cognitive sequelae of blast exposure, whereas the last two items were included to obtain global ratings on physical and mental health.

**References**

Eyres S, Carey A, Gilworth G, et al. Construct validity and reliability of the Rivermead Post Concussion Symptoms Questionnaire. Clin Rehabilitation. 2005;19(8):878-87. doi: 10.1191/0269215505cr905oa

King NS, Crawford S, Wenden FJ, et al. The Rivermead post concussion symptoms questionnaire: a measure of symptoms commonly experienced after head injury and its reliability. J Neurol. 1995;242(9):587-92. doi: 10.1007/BF00868811

Swiontkowski MF, Engelberg R, Martin DP, Agel J. Short musculoskeletal function assessment questionnaire: validity, reliability, and responsiveness. *J Bone Joint Surg.* (1999) 81:1245–60. doi: 10.2106/00004623-199909000-00006

Verfaellie M, Lafleche G, Spiro IIIA, et al. Chronic postconcussion symptoms and functional outcomes in OEF/OIF veterans with self-report of blast exposure. J Int Neuropsychol Soc 2013;19(1):1-10.

Ware JE Jr., Sherbourne C D. The MOS 36-item short-form health survey (SF-36). I. Conceptual framework and item selection. Med Care 1992;30(6):473-83.

Weathers FW, Litz BT, Keane TM, et al. The PTSD Checklist for DSM-5 (PCL-5). National Center for PTSD; 2013. Available online at: www.ptsd.va.gov
