# Supplementary figures and images for: A Distinct Metabolite Signature in Military Personnel Exposed to Repetitive Low-Level Blasts
Source: Front Neurol. 2022 Apr 7;13:831792. doi: 10.3389/fneur.2022.831792 (PMC9021419; doi:10.3389/fneur.2022.831792)

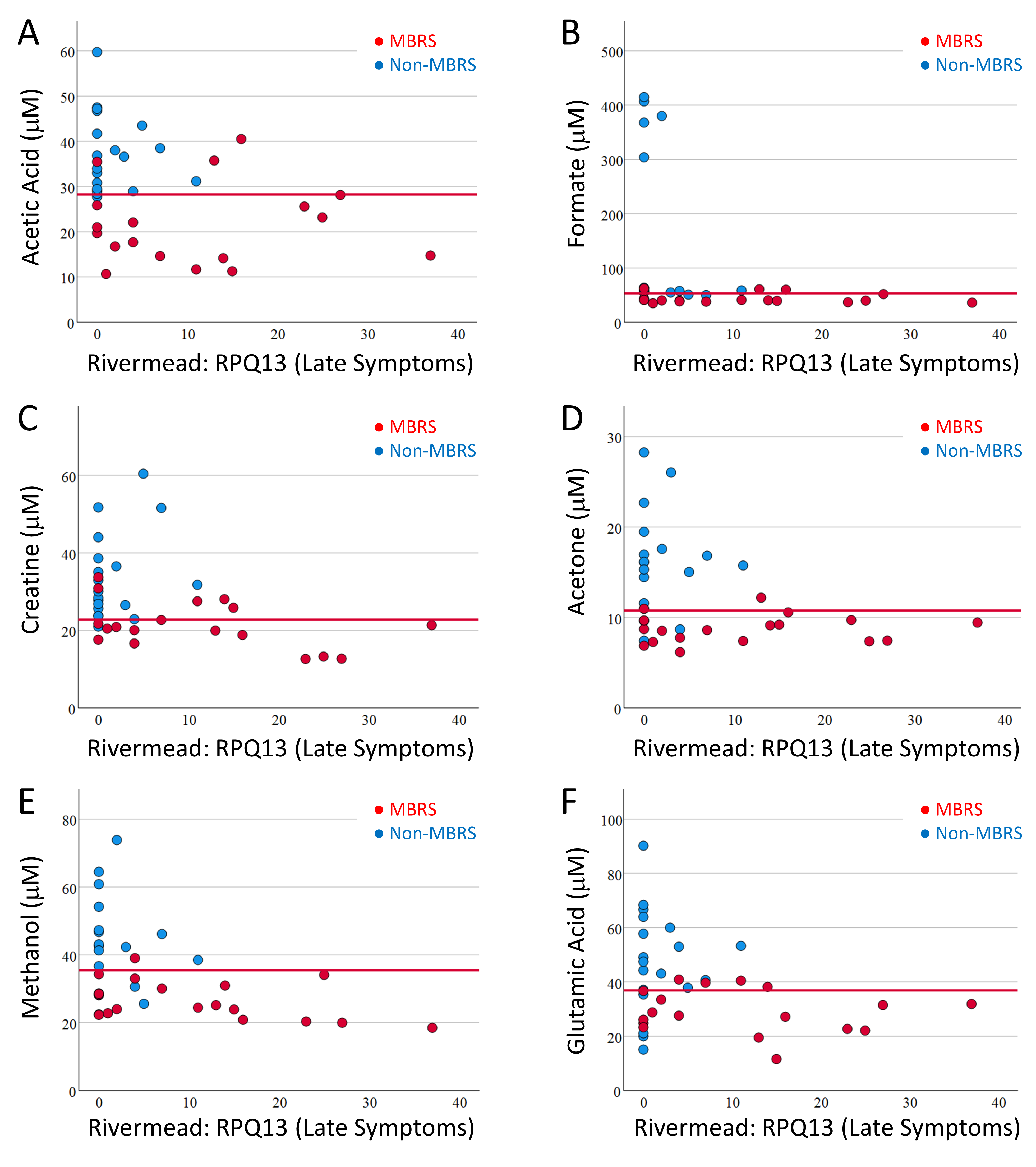

Supplement: Supplementary file 2 [file Image_1.TIF]

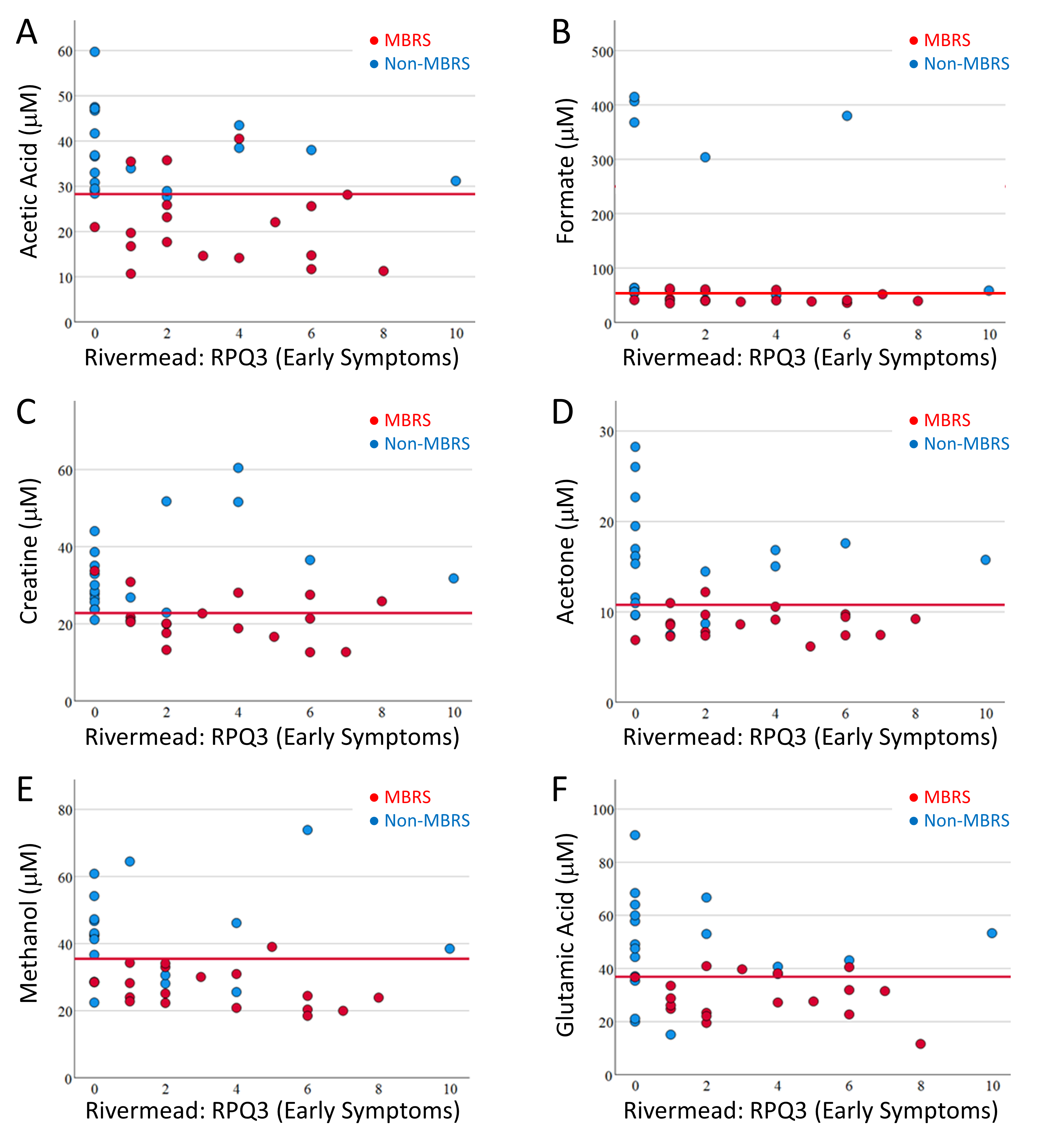

Supplement: Supplementary file 3 [file Image_2.TIF]

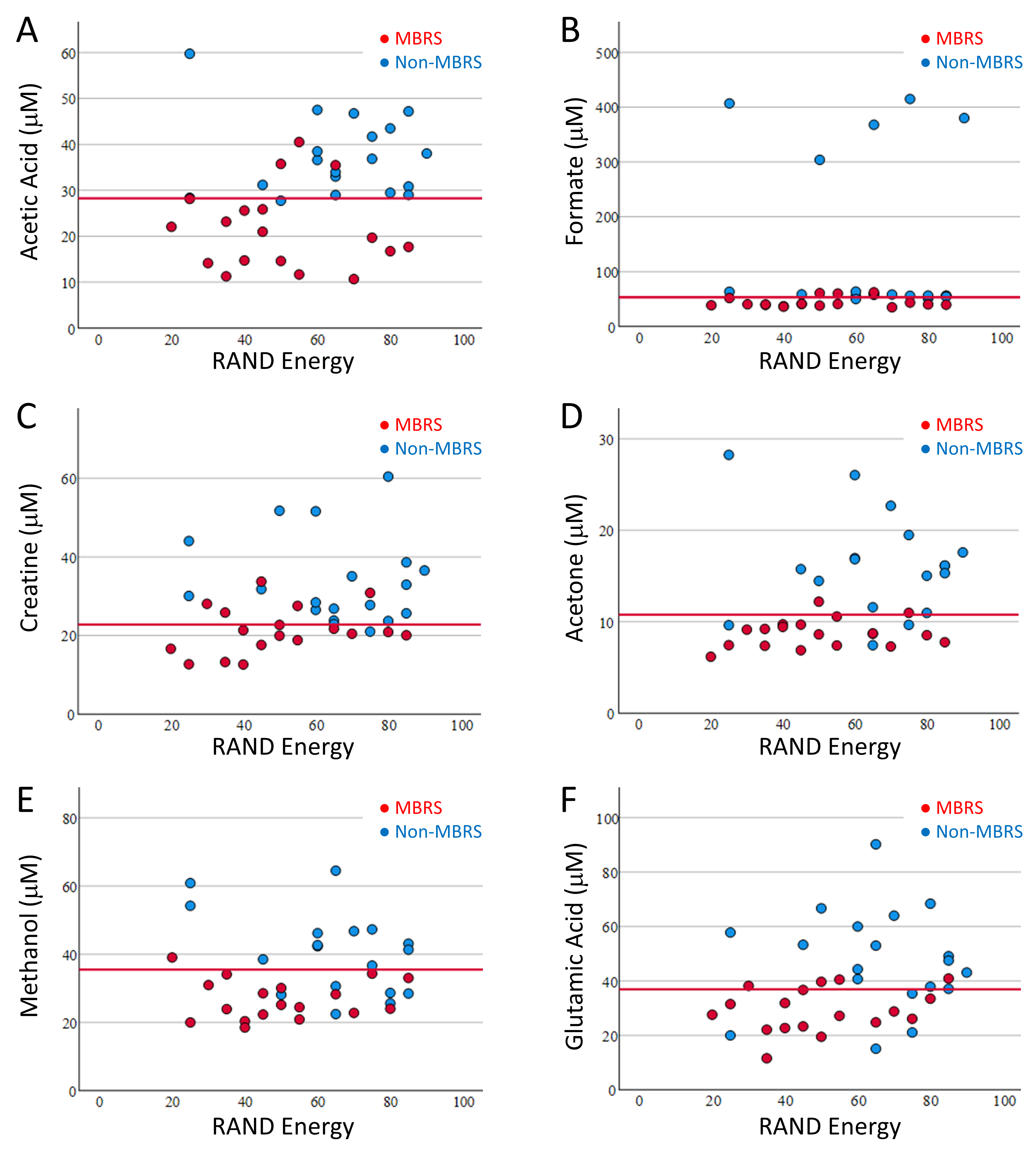

Supplement: Supplementary file 4 [file Image_3.TIF]
